# Engineering the Complex-Valued Constitutive Parameters of Metamaterials for Perfect Absorption

**DOI:** 10.1186/s11671-017-2048-2

**Published:** 2017-04-17

**Authors:** Pengwei Wang, Naibo Chen, Chaojun Tang, Jing Chen, Fanxin Liu, Saiqian Sheng, Bo Yan, Chenghua Sui

**Affiliations:** 1 Center for Optics & Optoelectronics Research, Department of Applied Physics, Zhejiang University of Technology, Hangzhou, 310023 China; 2 College of Electronic Science and Engineering, Nanjing University of Posts and Telecommunications, Nanjing, 210023 China; 3 National Laboratory of Solid State Microstructures, Nanjing University, Nanjing, 210093 China

**Keywords:** Metamaterials, Perfect absorbers, Anti-reflection coating, Light harvesting

## Abstract

We theoretically studied how to directly engineer the constitutive parameters of metamaterials for perfect absorbers of electromagnetic waves. As an example, we numerically investigated the necessary refractive index *n* and extinction coefficient *k* and the relative permittivity *ε* and permeability *μ* of a metamaterial anti-reflection layer, which could cancel the reflection from a hydrogenated amorphous silicon (α-Si:H) thin film on a metal substrate, within the visible wavelength range from 300 to 800 nm. We found that the metamaterial anti-reflection layer should have a negative refractive index (*n* < 0) for short-wavelength visible light but have a positive refractive index (*n* > 0) for long-wavelength visible light. The relative permittivity *ε* and permeability *μ* could be fitted by the Lorentz model, which exhibited electric and magnetic resonances, respectively.

## Background

In recent years, metamaterial-based electromagnetic wave perfect absorbers have attracted too much interest [1–3], owing to a wide variety of potential applications such as thermal emitters [4–6], sensor [7–10], photodetection [11, 12], solar cells or thermo-photovoltaics [13–17], and so on. In 2008, Landy et al. first experimentally demonstrated metamaterial perfect absorbers in the microwave region, by matching the effective wave impedance of metamaterials to the free-space wave impedance [18]. Their designed metamaterial perfect absorbers consist of two standard split-ring resonators connected by the inductive ring parallel to the split wire and a cut wire in a parallel plane separated by a substrate. This seminal work in the microwave regime has inspired many studies on metamaterial perfect absorbers in other frequency regimes including terahertz [19–21], mid-infrared [22, 23], near-infrared [24–26], and visible realm [27–29]. At the same time, there also have been many efforts to make metamaterial perfect absorbers as efficient and effective as possible, through carefully engineering their versatile properties like polarization independence [30–33], broad incident angle [34–37], broad bandwidth or multi-band [38–41], tunability [42–44], and flexibility [45, 46]. In most metamaterial perfect absorbers, there is always a three-layer design with a ground plane or metallic substrate supporting a dielectric and top metallic nanostructure [1]. The physical mechanisms behind many metamaterial perfect absorbers are essentially the same: the top metallic structure is utilized to couple to the incident electric field, and the anti-parallel currents between the two metallic layers are used to couple to the incident magnetic field. By tuning the metamaterial geometry, one could impedance match to free space at a desired frequency range [1]. For explaining the same phenomenon of perfect absorption of electromagnetic waves, there are also other physical models based on interference theory of reflected waves [47–50], such as transmission line theory [51–54], Fabry-Pérot, or other cavity resonances [55–62].

In many studies on metamaterial perfect absorbers, tailoring the morphology of the top metallic nanostructure has been explored widely as an effective way to obtain desired performances [1]. However, there are only few researches on directly engineering the complex-valued constitutive parameters of an artificial metamaterial for perfect absorbers of electromagnetic waves [63, 64]. For example, Ye et al. experimentally demonstrated a metamaterial perfect absorber in which the frequency-independent dispersion regions of both permittivity and permeability are stretched to an ultra-wide band from 0.5 to 2.5 GHz, and the real and imaginary parts of the complex constitutive parameters are precisely tuned to satisfy a modified model of a perfectly matched layer [63]. Long et al. deduced theoretically the critical coupling condition to achieve perfect absorption for thin-film absorbers of absorbing layer/spacer layer/substrate and identified numerically the key characteristics of the absorbing layer needed for perfect absorption at a given wavelength [64].

In this work, we will theoretically show how to directly engineer the complex-valued constitutive parameters of artificial metamaterials for perfect absorbers of electromagnetic waves. Specifically speaking, for achieving ultrawideband perfect absorption within the visible wavelength range from 300 to 800 nm, we have numerically investigated the necessary refractive index *n* and extinction coefficient *k*, and the relative permittivity *ε* and permeability *μ* of a metamaterial anti-reflection layer, coating a hydrogenated amorphous silicon (α-Si:H) thin film on a metal substrate. It is found that the metamaterial anti-reflection layer should have a negative refractive index (*n* < 0) for short-wavelength visible light, but has a positive refractive index (*n* > 0) for long-wavelength visible light. As the thickness of the metamaterial anti-reflection layer is increased, the absolute value of *n* and *k* will become smaller. However, they will become larger when the thickness of the α-Si:H thin film is increased. The relative permittivity *ε* and permeability *μ* could be fitted by the Lorentz model, exhibiting electric and magnetic resonances, respectively.

## Methods

**Fig. 1:**
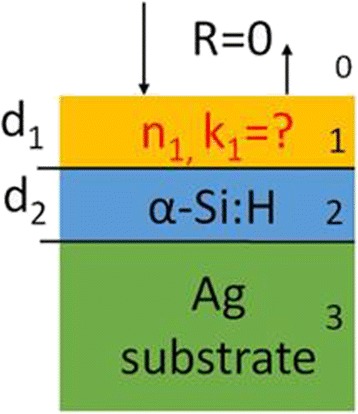
Schematic diagram of a perfect absorber of electromagnetic waves. The media labeled as 0, 1, 2, and 3 are air, metamaterial anti-reflection layer, hydrogenated amorphous silicon (α-Si:H) layer, and Ag substrate, respectively. The thicknesses of the anti-reflection and α-Si:H layers are *d*_1_ and *d*_2_, respectively

**Fig. 2:**
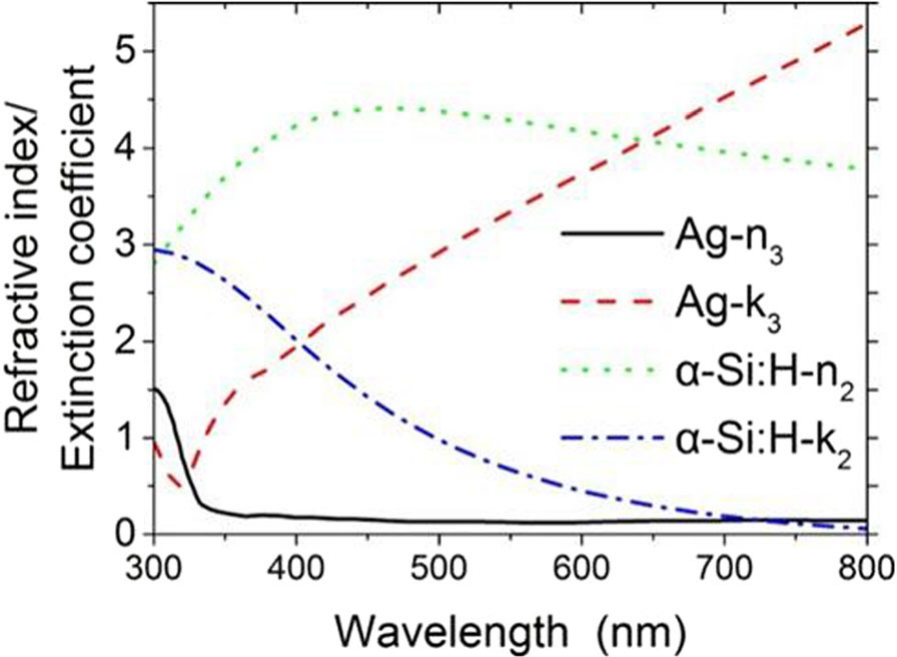
The refractive index *n* and extinction coefficient *k* of the Ag substrate and the hydrogenated amorphous silicon (α-Si:H) layer. The *black* (*solid*) *line* and the *red* (*dash*) *line* give the refractive index *n*_1_ and extinction coefficient *k*_1_ of Ag, respectively. The *green* (*dot*) *line* and the *blue* (*dash dot*) *line* give the refractive index *n*_2_ and extinction coefficient *k*_2_ of α-Si:H, respectively

The perfect absorber of electromagnetic waves investigated in this work is schematically shown in Fig. 1. Light is normally incident from air onto the perfect absorber, which consists of a metamaterial anti-reflection layer, and a hydrogenated amorphous silicon (α-Si:H) layer deposited on an Ag substrate. In our numerical calculations, the refractive index *n*_0_ of air is 1. The metamaterial anti-reflection layer has a complex refractive index of *n*_1_ *+ ik*_1_ and a thickness of *d*_1_. The α-Si:H layer has a complex refractive index of *n*_2_ *+ ik*_2_ and a thickness of *d*_2_. The Ag substrate has a complex refractive index of *n*_3_ *+ ik*_3_. In our numerical calculations, the used refractive index *n* and the extinction coefficient *k* of α-Si:H and Ag are presented in Fig. 2.

In the three-layer structure shown in Fig. 1, the reflection coefficient of light can be expressed as [64, 65]

1 $$ r=\frac{r_{01}+{r}_{123}{e}^{2 i{\beta}_1}}{1+{r}_{01}{r}_{123}{e}^{2 i{\beta}_1}}, $$

Where ${r}_{123}=\frac{r_{12}+{r}_{23}{e}^{2 i{\beta}_2}}{1+{r}_{12}{r}_{23}{e}^{2 i{\beta}_2}}$, ${\beta}_m=\frac{2\pi}{\lambda}{\tilde{n}}_m{\mathrm{d}}_m$, and *r*_*pq*_ = (*ñ*_*p*_ − *ñ*_*q*_)/(*ñ*_*p*_ + *ñ*_*q*_), which denotes the Fresnel reflection coefficients from medium *p* to medium *q. ñ*_*p*_ = *n*_*p*_ + i *k*_*p*_ is the complex refractive index of medium *p*. ${n}_p$ and ${k}_p$ are the refractive indexes and the extinction coefficient of medium *p*. The light reflectivity is given by $R={\left| r\right|}^2$. The light absorption can be written as $A=1- R$, because the substrate is metallic and there is no transmission.

## Results and discussion

**Fig. 3:**
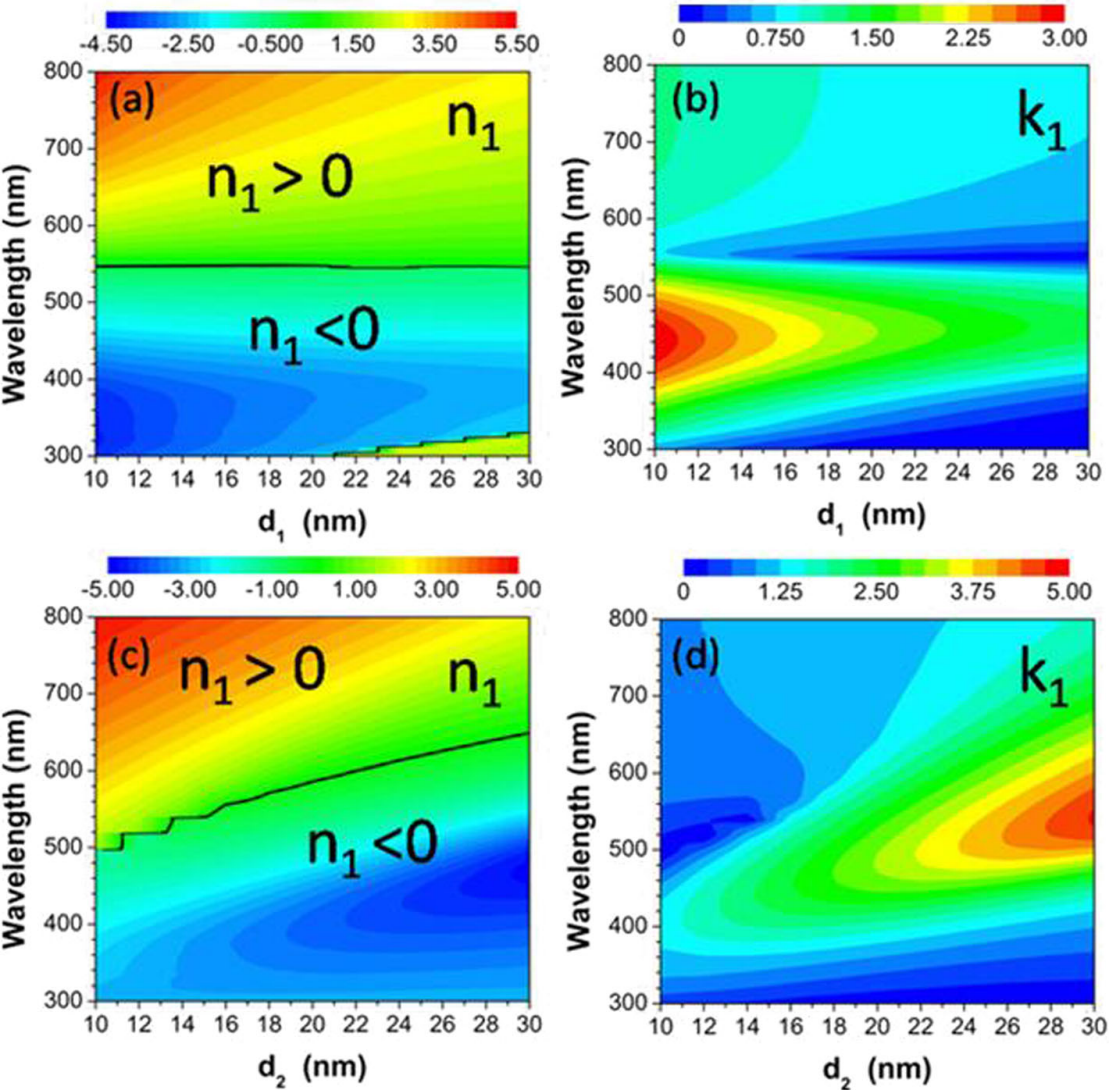
**a** The necessary refractive index *n*_1_ and **b** extinction coefficient *k*_1_ of the anti-reflection layer as a function of light wavelength and thickness *d*_1_ for achieving perfect absorption. The wavelength is varied from 300 to 800 nm. The thickness *d*_1_ of the anti-reflection layer is increased from 10 to 30 nm in steps of 2 nm. The thickness of the α-Si:H layer *d*_2_ = 15 nm. **c, d** The same as with **a** and **b**, but for thickness *d*_2_ increased from 10 to 30 nm, and *d*_1_ = 15 nm. The *black lines* in **a** and **c** indicate the boundaries at which the refractive index *n*_1_ is equal to zero

When the reflectivity *R* is equal to zero, the multi-layer structure shown in Fig. 1 can work as a perfect absorber. As the other physical and geometrical parameters are given, *R* is only a function of refractive index *n*_1_ and extinction coefficient *k*_1_ of the anti-reflection layer. By solving numerically the equation of *R* (*n*_1_, *k*_1_) = 0, one can obtain the values of *n*_1_ and *k*_1_ [64, 66]. In Fig. 3a, b, we show the solved values of *n*_1_ and *k*_1_ for achieving perfect absorption in the wavelength range from 300 to 800 nm, with the thickness *d*_1_ of the anti-reflection layer increased from 10 to 30 nm in steps of 2 nm, and the thickness *d*_2_ of the α-Si:H layer fixed to be 15 nm. It is clearly seen in Fig. 3a that the anti-reflection layer needs a kind of material having a negative refractive index (*n*_1_ < 0) for short wavelengths smaller than about 550 nm. However, the anti-reflection layer needs another kind of material having a positive refractive index (*n*_1_ > 0) for longer wavelengths. As the thickness *d*_1_ is increased, the absolute value of *n*_1_ will become smaller, and the value of *k*_1_ will also become smaller, as exhibited in Fig. 3b. We have also investigated the effect of thickness *d*_2_ on the necessary refractive index *n*_1_ and extinction coefficient *k*_1_ for achieving perfect absorption. As clearly seen in Fig. 3c, d, both the absolute value of *n*_1_ and the value of *k*_1_ will become larger with increasing thickness *d*_2_, which is in contrast to the case in Fig. 3a, b.

Here, we discuss the physical mechanism that underlined the perfect absorber of light waves. In order to obtain perfect absorption or zero reflection, the two terms (${r}_{01}$ and ${r}_{123}{e}^{2 i{\beta}_1}$) in Eq. (1) should have the same amplitude but opposite phase, from which one can deduce two equations to define the condition for perfect absorption [64]: $4\pi {k}_1{d}_1/\lambda = In\left({R}_{123}/{R}_{01}\right)$,$4\pi {n}_1{d}_1/\lambda =\pi +{\varphi}_{01}-{\varphi}_{123}$, where ${R}_{01}$(${R}_{123}$) and ${\varphi}_{01}$(${\varphi}_{123}$) denote the amplitude and the phase of ${r}_{01}$(${r}_{123}$), respectively. When both equations are satisfied, completely destructive interference will happen. As a result, reflection is reduced to zero, and perfect absorption is achieved.

In many cases, the permittivity and permeability are also often used to exhibit the constitutive parameters of electromagnetic materials. So, in order to further understand the conditions of perfect absorption, the relative permittivity *ε* and permeability *μ* of the metamaterial anti-reflection layer are also investigated. We can calculate numerically the real and imaginary parts (*ε*_*r*_, *ε*_*i*_, *μ*_*r*_, *μ*_*i*_) of *ε* and *μ*, using the following relation of constitutive parameters in Eq. (2)

2 $$ \begin{array}{c}\hfill n = {n}_1 + i{k}_1\hfill \\ {}\hfill \begin{array}{c}\hfill n = \pm \sqrt{\varepsilon \mu}\hfill \\ {}\hfill \varepsilon = {\varepsilon}_r + i{\varepsilon}_i\hfill \\ {}\hfill \mu = {\mu}_r + i{\mu}_i\hfill \end{array}\hfill \end{array} $$

**Fig. 4:**
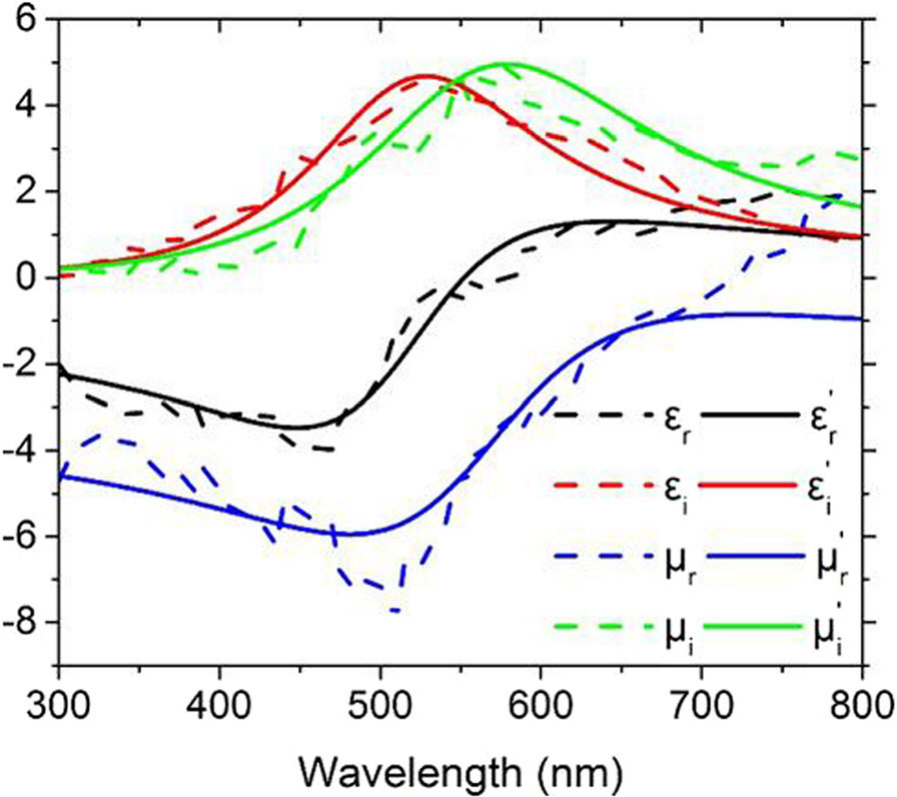
The relative permittivity *ε* and permeability *μ* of the metamaterial anti-reflection layer for achieving perfect absorption, with the thickness of the anti-reflection layer *d*_1_ = 15 nm and the thickness of the α-Si:H layer *d*_2_ = 30 nm. The *dashed lines* give the numerically calculated values of the real and imaginary parts (*ε*_*r*_, *ε*_*i*_, *μ*_*r*_, *μ*_*i*_) of *ε* and *μ*, and the *solid lines* give the corresponding results (*ε*_*r*_^*,*^, *ε*_*i*_^*,*^, *μ*_*r*_^*,*^, *μ*_*i*_^*,*^) fitted through the Lorentz model

The choice of sign “±” in Eq. (2) must ensure that *k*_1_ is positive because electromagnetic waves propagating in a passive medium always suffer a loss. As an example, the dashed lines in Fig. 4 present the calculated values of the real and imaginary parts of *ε* and *μ*, for the thickness of the anti-reflection layer *d*_1_ = 15 nm and the thickness of the α-Si:H layer *d*_2_ = 30 nm. The numerical results can be fitted approximately through the Lorentz model, in which the complex permittivity and permeability can be represented as

3 $$ \begin{array}{c}\hfill {\varepsilon}^{\prime }={\varepsilon}_1+\frac{\omega_p^2}{\omega_{a1}^2-{\omega}^2- i{\omega}_{c1}\omega}\hfill \\ {}\hfill {\mu}^{\prime }={\mu}_1+\frac{\omega_p^2}{\omega_{a2}^2-{\omega}^2- i{\omega}_{c2}\omega}\hfill \end{array} $$

Here, *i* is the imaginary unit, ${\omega}_p$ is the plasma frequency, ${\omega}_{a1},{\omega}_{a2}$ are the center frequencies of the oscillators,${\omega}_{c1},{\omega}_{c2}$ are the damping frequencies, and ${\varepsilon}_1,{\mu}_1$ are the static permittivity and permeability at infinite frequency, respectively. The solid lines in Fig. 4 show the fitted results by Eq. (3), with the corresponding parameters ${\varepsilon}_1=-3.9,{\mu}_1=-1.5,{\omega}_p=4.6*{10}^{15} Hz,{\omega}_{a1}=3.32*{10}^{15} Hz,{\omega}_{a2}=3.62*{10}^{15} Hz,{\omega}_{c1}=1.3*{10}^{15} Hz,{\omega}_{c2}=1.26*{10}^{15} Hz$. It is clearly seen in Fig. 4 that the metamaterial anti-reflection layer should have both electric and magnetic resonances.

Finally, we would like to discuss the possibility of realizing the designed perfect absorber of visible light. For the perfect absorber, the complex permittivity and permeability of the anti-reflection layer must exhibit electric and magnetic resonances, respectively, which are described by the Lorentz model. When the real parts of the complex permittivity and permeability are simultaneously negative, the refractive index of the anti-reflection layer will have a negative value; otherwise, it will have a positive value. It is well-known that artificial metamaterials can exhibit Lorentz dispersion, whose constitutive parameter (either the permittivity or the permeability) obeys the *K*-*K* relations. In fact, with the fast development of nanofabrication technology, metamaterials with both electric and magnetic resonances have been fabricated successfully to realize negative refractive index at optical frequencies, which are composed of pairs of parallel metallic nanorods [67, 68]. Recently, an experimental work demonstrated that the real and imaginary parts of the complex constitutive parameters of metamaterials consisting of metallic split-ring resonator and rod could be deliberately controlled to produce a wide-band perfect absorption in gigahertz [63]. Based on the advancement of optical lumped nanocircuits, this approach may be extended to infrared and optical bands, as mentioned in the experimental work.

## Conclusions

In this work, we have theoretically shown how to directly engineer the complex-valued constitutive parameters of artificial metamaterials for perfect absorbers of electromagnetic waves. Specifically speaking, for achieving ultra-wideband perfect absorption within the visible wavelength range from 300 to 800 nm, we numerically investigated the necessary refractive index *n* and extinction coefficient *k*, and the relative permittivity *ε* and permeability *μ* of a metamaterial anti-reflection layer, coating a hydrogenated amorphous silicon (α-Si:H) thin film on a metal substrate. The numerical results show that the metamaterial anti-reflection layer has a negative refractive index (*n* < 0) for short-wavelength visible light, but has a positive refractive index (*n* > 0) for long-wavelength visible light. As the thickness of the metamaterial anti-reflection layer is increased, the absolute value of *n* and *k* will become smaller. However, they will become larger when the thickness of the α-Si:H thin film is increased. It is also found that such a metamaterial anti-reflection layer should have both electric and magnetic resonances, and its permittivity *ε* and permeability *μ* can be approximately fitted by the Lorentz model. We hope that these results presented in this work could provide another approach to design perfect absorbers of electromagnetic waves, with the fast development of nanofabrication technology in metamaterials.
